# Speed and accuracy of visual image discrimination by rats

**DOI:** 10.3389/fncir.2013.00200

**Published:** 2013-12-18

**Authors:** Pamela Reinagel

**Affiliations:** Section of Neurobiology, Division of Biological Sciences, University of California at San DiegoLa Jolla, CA, USA

**Keywords:** decision making, sequential decision, speed–accuracy trade-off, rodent vision, visual behavior, perceptual decision, choice

## Abstract

The trade-off between speed and accuracy of sensory discrimination has most often been studied using sensory stimuli that evolve over time, such as random dot motion discrimination tasks. We previously reported that when rats perform motion discrimination, correct trials have longer reaction times than errors, accuracy increases with reaction time, and reaction time increases with stimulus ambiguity. In such experiments, new sensory information is continually presented, which could partly explain interactions between reaction time and accuracy. The present study shows that a changing physical stimulus is not essential to those findings. Freely behaving rats were trained to discriminate between two static visual images in a self-paced, two-alternative forced-choice reaction time task. Each trial was initiated by the rat, and the two images were presented simultaneously and persisted until the rat responded, with no time limit. Reaction times were longer in correct trials than in error trials, and accuracy increased with reaction time, comparable to results previously reported for rats performing motion discrimination. In the motion task, coherence has been used to vary discrimination difficulty. Here morphs between the previously learned images were used to parametrically vary the image similarity. In randomly interleaved trials, rats took more time on average to respond in trials in which they had to discriminate more similar stimuli. For both the motion and image tasks, the dependence of reaction time on ambiguity is weak, as if rats prioritized speed over accuracy. Therefore we asked whether rats can change the priority of speed and accuracy adaptively in response to a change in reward contingencies. For two rats, the penalty delay was increased from 2 to 6 s. When the penalty was longer, reaction times increased, and accuracy improved. This demonstrates that rats can flexibly adjust their behavioral strategy in response to the cost of errors.

## INTRODUCTION

The temporal dynamics of decision making have been most thoroughly studied using the random dot motion task, in which a number of randomly positioned dots move coherently in one of two directions (the signal), while a number of other randomly positioned dots move in random directions (the noise). Thus information about the direction of coherent motion is embedded in noise, and averaging over time improves the signal-to-noise ratio of the sensory information available in the physical stimulus. When human and primate subjects perform this task, subjects wait longer to respond when the stimuli are less coherent (more ambiguous), and there is a trade-off between accuracy and speed (Palmer et al., 2005). Speed–accuracy trade-off in primate vision has been the subject of a rich experimental and theoretical literature (Britten et al., 1996; Shadlen and Newsome, 1996, 2001; Leon and Shadlen, 1998; Kim and Shadlen, 1999; Gold and Shadlen, 2001, 2007; Hastie and Dawes, 2001; Roitman and Shadlen, 2002; Glimcher, 2003; Huk and Shadlen, 2005; Palmer et al., 2005; Bogacz et al., 2006, 2009; Churchland et al., 2008; Ratcliff and McKoon, 2008; Kahneman, 2011; Liu and Pleskac, 2011; Drugowitsch et al., 2012).

Compared with primates, little is known about the trade-off of speed and accuracy in sensory decisions by rodents. In the past decade, studies have begun to address this question in rodents using olfactory (Uchida and Mainen, 2003; Abraham et al., 2004; Kepecs et al., 2006, 2007; Rinberg et al., 2006; Uchida et al., 2006; Felsen and Mainen, 2008, 2012) and auditory (Jaramillo and Zador, 2011; Sanders and Kepecs, 2012; Brunton et al., 2013) tasks. For the case of rodent vision, it was recently shown that that when rats perform the random dot visual motion task, accuracy improves with viewing time and viewing time increases with the discrimination difficulty (Reinagel, 2013). The improvement in accuracy with reaction time required the presence of the ongoing motion stimulus. This raised the question whether this improvement with viewing time required that the stimulus be dynamically updated with new independent evidence for the decision (as is the case with random dot motion), or whether the same would hold true when the stimulus was well above-threshold and static. In motion discrimination, the increase in reaction time with difficulty was smaller than expected for integration to a bound, and more resembled the responses of humans and monkeys when given a deadline or instructed to prioritize speed over accuracy. Moreover, the increase in reaction time with difficulty was found even under conditions (after stimulus offset) when the delay impaired rather than improved reward outcome. Thus the dependence of reaction time on difficulty could reflect confidence (Kepecs et al., 2008; Kiani and Shadlen, 2009) rather than sensory integration time. It remained unclear, then, whether rats have the capacity to prioritize accuracy any more highly in this task, and whether doing so would result in a change in speed.

To address these questions, this study describes the relationship between reaction time and accuracy in the responses of rats discriminating between high-contrast static visual images. The visual similarity of the image pair was varied parametrically by image morphing. Rats’ ability to modulate reaction time in response to task demands was tested by changing the duration of the error penalty.

## MATERIALS AND METHODS

### ANIMALS

Twelve female Long-Evans rats (Harlan) were water restricted and trained to perform visual tasks for water reward (Meier et al., 2011). Subjects began training at age p30 for 2 h/day 7 days a week. Subjects performed 500–1500 trials per day, and received water in 50% of trials when performing at chance. No supplemental water (outside of the task) was given at any time, but carrots were given after each training session. During training sessions subjects had free access to return to the home cage at any time; thus they had access to food during periods of water consumption. On this protocol, all subjects maintained normal growth curves (within 5% of published values for unrestricted food and water). Between training sessions, subjects were pair-housed with enrichment (chew toys, PVC tubes). Subjects were housed in a reverse 12 h light/dark cycle and were trained and tested in the housing environment during the dark cycle. All 12 rats that began the study learned the task and completed the study. The total training time in calendar days from naive animal to beginning the testing phase (shaping steps 1–5) ranged from 29 to 108 days (56.1 ± 26.3, mean ± SD), corresponding to ages between p59 and p138. The calendar days required to complete the testing period (step 6) ranged from 20 to 42 days (27.4 ± 5.9, mean ± SD). All procedures were performed with the approval and under the supervision of the UCSD IACUC, within an ALAAC accredited animal facility. The image discrimination task was described previously (Clark et al., 2011). The reaction time data reported here were collected from the pre-lesion and un-lesioned subjects of that earlier study.

### APPARATUS

**FIGURE 1:**
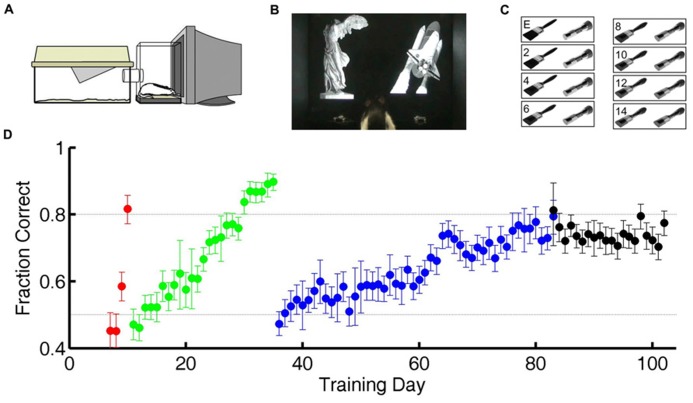
**Training and testing paradigm.** **(A)** Diagram of cage-attached operant conditioning chamber. **(B)** One of the subjects in this study in the operant chamber performing the statue-shuttle image discrimination (shaping step 4). **(C)** The exemplar image pair E and examples of the intermediate morph pairs for the flashlight–paintbrush image discrimination used in the testing phase (shaping step 6). **(D)** Example learning curve for one subject showing performance as a course of training from naïve to study completion. Training day indicates number of calendar days since initiating training. Chance performance is 0.5 (lower dotted line). In the first two shaping steps (acclimation to apparatus, shaping steps 1–2, days 1–6 in this case) all responses are valid, so performance is undefined (not plotted). For all subsequent shaping steps, each symbol shows the average performance on one task over one training day. Error bars show 95% binomial confidence intervals. Color indicates task: go to statue (shaping step 3, *red*), discriminate statue from shuttle (shaping step 4, *green*), discriminate flashlight from paintbrush exemplars (shaping step 5, *blue*), or discriminate flashlight from paintbrush including exemplars and morph probe trials (shaping step 6, *black*). Subjects were automatically graduated to the next task when performance exceeded 80% (upper dotted line) for at least 200 trials, and graduated from the final task when each morph level had been tested exactly 150 times.

The training apparatus and software are described in detail in Meier et al. (2011). Briefly, training occurred in a small, clear Lucite training chamber with a CRT monitor visible through one wall (**Figure 1A**). The CRT monitor (NEC FE992-19, 100 Hz, 1024 × 768 resolution) was linearized with a minimum, mean, and maximum luminance of 4, 42, and 80 cd/m^2^, respectively (Colorvision, spyder2express). From the position of the center request port, the monitor was about 10 cm from the rat’s eye and subtended 104° of visual angle (0.1 degrees/pixel). Images were displayed immediately above the two response ports and subtended about 35° of visual angle (shaping steps 3 and 4) or 20° (shaping steps 5 and 6) in their maximum dimension. A central “request” port was located near the bottom of the display wall; two “response” ports were located 90 mm left and right of this. Request and response ports were triggered by licking a water tube, which was detected when the rat’s tongue broke an infrared beam. Lick times were the only recorded behavioral output; nose position was not separately monitored. The volume of water drop delivered for reward was determined by the duration of valve opening (50 ms) on a low-pressure water line. Due to pressure variations, the precise volume varied from day to day, but was matched across the ports.

In this apparatus, response required locomotion, which introduces a time and effort cost for the rat. This may increase the rats’ prioritization of accuracy in our tasks overall. Although long and variable response times might have overwhelmed any systematic differences between stimulus and reward conditions, we found that such differences could still be resolved. Nevertheless, other response modalities can be executed and detected more quickly, and could be used to place tighter bounds on the time required for rats to make sensory decisions.

### SHAPING

In preliminary shaping, subjects moved through four shaping steps (**Table 1**) to acquire a two alternative forced-choice (2AFC) visual discrimination between static grayscale photographic images of two real world objects (a statue and a space shuttle; **Figure 1B**). In this and all subsequent steps, each trial was initiated by the subject by licking a central request port, which caused the two images to appear on the screen, one above each response port. The rewarded (S+) stimulus was randomly assigned to either the left (L) or right (R) side of the screen, and the unrewarded (S-) stimulus to the other side. The two images were large and high contrast, and were matched in luminance, size, contrast, and orientation. The images persisted until the subject licked a response port (L or R), with no time limit. Responses at the port co-localized with the S+ stimulus were rewarded with water delivered at the same location with <10 ms delay, after which the subject could immediately initiate a new trial. Responses at the port co-localized with the S- stimulus were penalized with a timeout of 2–8 s before a new trial could be initiated. After each correct trial, the S+ stimulus was assigned to L or R side with equal probability. After an error trial, however, there was a fixed probability (0.25–0.5) of entering a correction trial instead, in which case the S+ stimulus was deterministically placed at the port opposite the previous trial’s response. This method was highly successful in helping rats overcome bias (overall preference for one response port over the other) as well as perseveration (preference to return to the most recently visited or recently rewarded port) over months of automated training and testing. However it alters the statistics of the task in trials after errors. Therefore only trials after correct trials are analyzed here.

**Table 1:** Details of shaping sequence for task acquisition.

Shaping step	Description	Days to complete (min–max)
1. Free drinks	Water released at any port when triggered by licking, and also un-triggered at random times.	0–4
2. Earned drinks	Water at any port when triggered by licking only; requires rotating among all three ports.	0–9
3. Approach visual target, 2AFC	Upon request (licking unrewarded center port), S+ (statue) image appears over one response port; responses at S+ rewarded with water, response on other side (no image) penalized with timeout.	4–11
4. Visual discrimination, 2AFC	Upon request S+ (statue) image appears over one response port and matched S- (space shuttle) over the other. Responses at S+ rewarded with water, response at S- penalized with timeout.	16–43
5. Exemplar discrimination, 2AFC	Same as previous, but S+ is now either flashlight or paintbrush, and S- is the other image of this pair.	29–108
6. Testing: exemplar and probes, 2AFC	Same as previous, but 20% of trials are probes with morphed intermediates between S+ and S-.	50–141

Reward magnitude was not varied in this study. Penalty time out duration was empirically adjusted for each rat to discourage guessing, while avoiding excessive subject frustration as judged by quitting. The penalty duration was always fixed for each rat within a training session. All rats began with a penalty duration set at 2 s. For seven of the subjects, this value was never changed over the course of training and testing. For five subjects, the penalty was increased by steps of 2 s, waiting on average 5000 trials between adjustments, up to a maximum of 8 s.

After mastering the first 2AFC visual discrimination (shaping step 4), subjects learned a second visual discrimination between two novel images (a paintbrush and a flashlight), one of which was assigned to be the S+ stimulus for each rat (shaping step 5). Subjects were trained on this “exemplar” discrimination until performance exceeded 80% accuracy for at least 200 trials (**Figure 1D**) before entering the test phase (shaping step 6). After completing shaping step 5, animals appear to make stereotypical head and body movements toward one or the other response port as soon as they leave the center port (see Video S1 in Supplementary Material), but head and eye movements were not tracked during training or testing.

### TESTING

In the test phase, subjects continued to be tested on the exemplar discrimination in 80% of trials; later analysis confirmed that performance on the exemplar pair was stationary for the duration of the test phase. In the remaining 20% of trials (interleaved), subjects were presented with a pair of images of parametrically varied similarity, obtained by morphing between the S+ and S- exemplar images (**Figure 1C**). In these probe trials, subjects were rewarded for responding at the port co-localized with the stimulus that was closer to S+ of the two images. A previous study had shown that rats were unlikely to be relying on any one local cue to discriminate the morphs, because results were qualitatively similar if any quadrant of the image was masked in both images of the pair (Clark et al., 2011). The order of probe trial types was pseudorandom with the constraint that each of the 14 non-exemplar difficulty levels had to be presented once before any one difficulty level could be repeated. This procedure ensured that data for probe trials accrued at the same rate for every difficulty level. Each rat continued the test phase until each probe type was tested exactly 150 times. During testing the penalty duration was fixed at 2 s for all rats.

### ANALYSIS

The data for each trial in the test phase consist of: which specific image pair was shown (selected independently each trial); on which side the rewarded target appeared (selected independently each trial); the time of subject-initiated stimulus request; the latency from stimulus onset to response; and the outcome of the trial (correct/reward or error/timeout). Data analysis was performed using custom programs written in Matlab (Mathworks, Natick, MA, USA).

**FIGURE 2:**
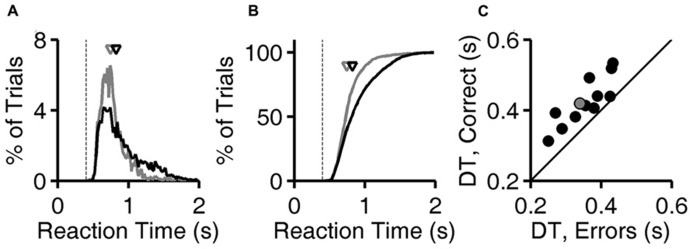
**Longer latencies in correct trials.** **(A)** Distribution of reaction times for error trials (gray) and correct trials (black) for exemplar discriminations for one subject (same subject as **Figure 1D**). Normalized probability distributions are shown, but there were about five times more correct trials than error trials. Arrows indicate median latencies of the distributions (0.743 s for errors, 0.823 s for correct responses). Dashed line is the minimum reaction time this subject showed in any trial or task (0.403 s). **(B)** Cumulative distributions of reaction time, the integrals of curves shown in panel **(A)**. **(C)** Median decision time in error trials (*x*-axis) and in correct trials (*y*-axis) for each subject (*N* = 12), for exemplar discriminations in the test phase. The example subject used in panels **(A,B)** is highlighted (gray). Symbols are above the identity line (diagonal) if correct trials had longer median reaction time.

**FIGURE 3:**
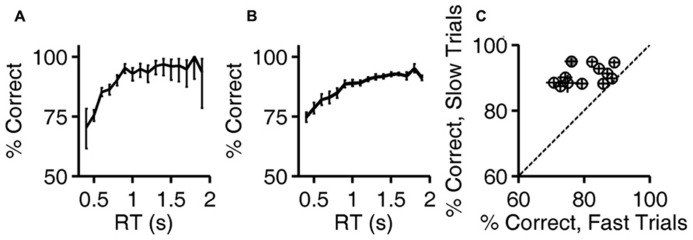
**Accuracy improves with reaction time.** **(A)** Accuracy of exemplar discrimination as a function of reaction time for a single subject (same rat as **Figures 1D** and **2A,B**); error bars show the 95% binomial confidence intervals. **(B)** Accuracy of exemplar discrimination as a function of reaction time averaged over all *N* = 12 rats; error bars show SEM over the population. **(C)** Accuracy on exemplar discrimination in fast trials vs. in slow trials in the test phase. Each symbol represents data from a single rat, and error bars show 95% binomial confidence intervals. The example subject used in **(A,B)** is highlighted (gray). Symbols are above the identity line (diagonal) if slow trials had higher accuracy.

Calculations are based on all valid trials (after excluding trials after errors) of the indicated type in the relevant testing block. In **Figures 2A,B**, reaction time distributions were computed from 4583 (correct) and 1483 (error) trials. Same data were used to compute **Figure 3A**. In **Figure 2C**, each point was computed from an average of 5815 correct trials (range 4583–7031) and 952 error trials (range 516–1483). The same data were used to compute the *N* = 12 curves that underlie the average curve in **Figure 3B**, and to compute the values per rat plotted in **Figure 3C**.

**FIGURE 4:**
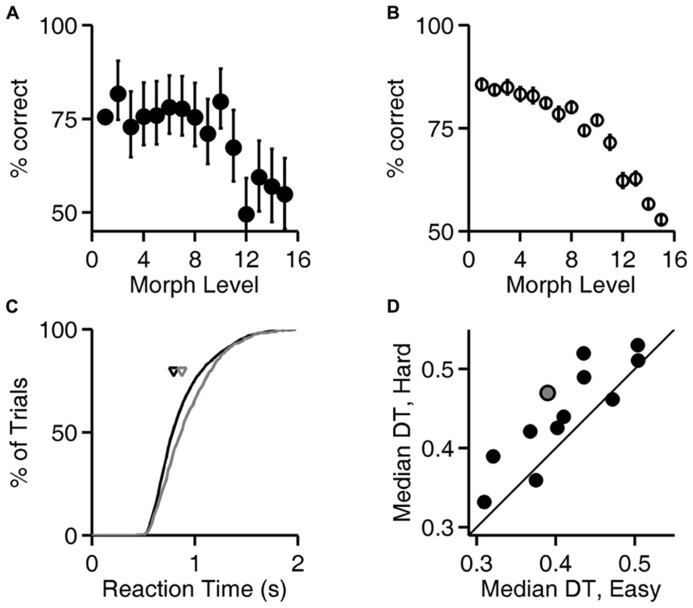
**Reaction time increases with trial difficulty.** **(A)** Performance (% correct responses) as a function of stimulus ambiguity (morph level) for one rat (cf. **Figures 1D, 2A,B**, and **3A**). Error bars show 95% binomial confidence intervals. **(B)** Average performance of all 12 subjects as a function of the similarity of the two images discriminated. Error bars show SEM over the population of *N* = 12 subjects. (Data re-analyzed from Clark et al., 2011). **(C)** Cumulative distribution of reaction time for the subject analyzed in panel **(A)**, for the easiest (level 1, black curve) and hardest (levels 12–15, gray curve) trials. Arrows indicate the median latencies of the two distributions (0.793 vs. 0.873 s). This subject’s minimum RT (estimated sensorimotor delay) was 0.403 s. **(D)** Median decision time (DT; reaction time minus sensorimotor delay) for easiest vs. hardest trial types for all *N* = 12 rats; data for the subject shown in panels **(A,C)** is highlighted in gray. Symbols above the diagonal identity line (*N* = 10/12) indicate a subject that takes more time to respond on harder discriminations.

**FIGURE 5:**
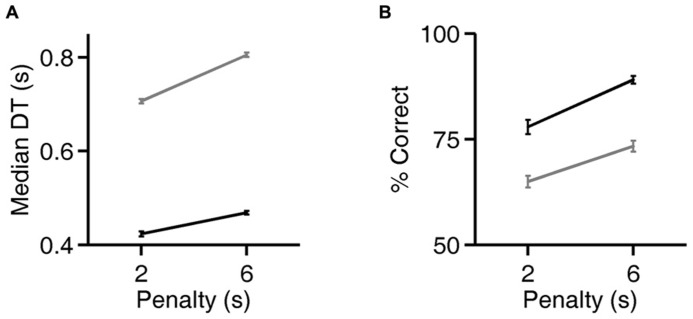
**Rats can flexibly prioritize either speed or accuracy depending on task contingencies.** **(A)** Median decision time (DT) on the exemplar discrimination, as a function of the duration of the error penalty for two rats (black, gray). Both correct trials and errors had higher DT when penalty was longer; all trials are included in the analysis shown. Error bars show standard errors of the means (SEM). DT is defined as the observed reaction time (RT) minus the rat’s estimated motor latency (lifetime minimum reaction time). This subtracted constant was 0.403 (black) or 0.363 s (gray). For raw reaction time values see **Figure 6**. **(B)** Accuracy of discriminations in the same trials analyzed in panel **(A)**. Error bars show 95% binomial confidence intervals.

**FIGURE 6:**
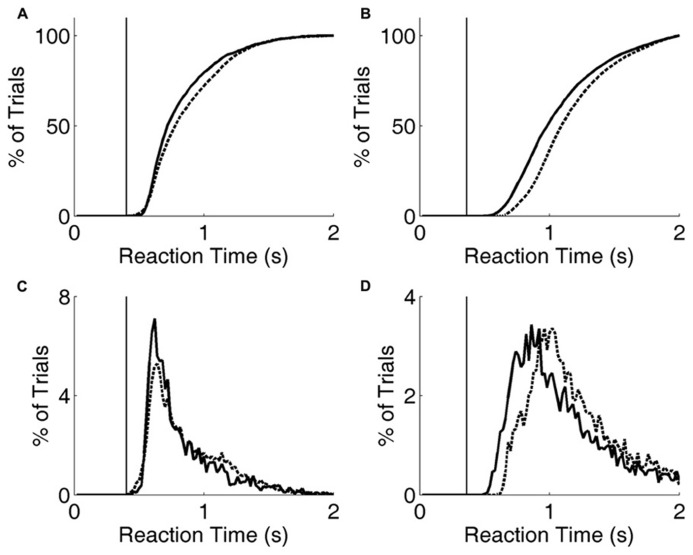
**Reaction time distribution shifts with penalty change.** **(A)** Cumulative probability distribution of reaction times for rat performing the exemplar discrimination (black lines in **Figure 5**), with short penalty (solid curve) or long penalty (dashed curve). Median reaction time increased from 0.724 to 0.787 s. The rat’s lifetime minimum reaction time is indicated by the thin vertical line. **(B)** Cumulative probability distribution of reaction times for a different rat performing a more difficult image discrimination (gray lines in **Figure 5**). Median reaction time increased from 0.993 to 1.102 s. The rat’s lifetime minimum reaction time is indicated by the thin vertical line. **(C)** Raw reaction time distributions corresponding to data of panel **(A)**. **(D)** Raw reaction time distributions corresponding to data of panel **(B)**.

**Figure 4A**, analysis of level 1 (exemplar discrimination) was based on 6126 valid trials; other levels (morph probe trials) were based on an average of 110 valid trials each (range 101–119). **Figure 4B** represents result from *N* = 12 rats, number of trials per condition similar to the example in **Figure 4A**. Cumulative probability in **Figure 4C** is based on 6126 (easy) vs. 442 (hard) trials. Median decision times in **Figure 4D** are based on an average of 6894 trials for the easy condition (range 6126–7627) and an average of 490 trials for the hard condition (range 442–539). Results in **Figures 5** and **6** are based on an average of 4089 valid trials per condition (range 2320–4807).

## RESULTS

Twelve Long-Evans rats were trained to discriminate between grayscale photographs of two perceptually similar objects – a flashlight and a paintbrush – in a self-paced 2AFC operant conditioning paradigm (**Figures 1A–D**; Materials and Methods; **Table 1**; Video S1 in Supplementary Material). After performance was asymptotic on this “exemplar” discrimination, subjects began the testing phase. During testing, the exemplar discrimination was tested in 80% of trials; the remaining 20% of trials were probe trials in which the discriminated images were rendered more similar by morphing between the exemplar images (**Figure 1C**). In probe trials, subjects were rewarded for selecting the image that more closely resembled the learned target.

### REACTION TIME IS LONGER IN CORRECT TRIALS

For each trial, the “reaction time” is defined here as the time between voluntary initiation of the trial (lick at center, at which time images appear) and the time of the subject’s response (lick at left or right response port, at which time the images disappear and reward or penalty occurs). The probability distribution of reaction times for exemplar discriminations is shown for both correct trials and for error trials for one rat (**Figures 2A,B**). For this subject, shorter reaction times (0.5–1.0 s) are more frequent among error trials, while long reaction times (1.0–1.5 s) are more frequent among correct trials. The median reaction time was longer in correct trials than error trials for this subject (arrows in **Figures 2A,B**), and this was the case for all 12 subjects (*P* < 10^-^^3^ by Wilcoxon signed rank test).

The minimum reaction time of a given subject across all trials and all visual 2AFC tasks (dashed line, **Figures 2A,B**) places an upper bound on the time required for the center-port to response-port motor response for that subject. The minimum reaction time was stable over time and tasks for a given subject, probably representing occasional pure motor responses (fast guessing). It ranged from 0.323 to 0.413 s across subjects. During visual tasks, responses were rarely as fast as the rat’s estimated motor delay.

The “decision time” in each trial is operationally defined here as the reaction time minus the subject’s sensory/motor delay as defined above. The median decision time (DT) for correct trials was longer than in error trials for all 12 subjects (**Figure 2C**; *P* < 10^-^^3^ by Wilcoxon signed rank test). Note that the DT differs from reaction time only by the subtraction of the same constant from both values for any given point, and therefore does not affect the sign or magnitude of the difference between compared conditions within subject.

### DEPENDENCE OF ACCURACY ON REACTION TIME

The fact that reaction times tended to be longer in correct trials implies that accuracy (% correct) was higher in trials with longer reaction times. The relationship between reaction time and accuracy on exemplar trials is shown for an example subject in **Figure 3A**. For this rat, performance improved with reaction time over the range of 0.5–1.2 s, beyond which there was no improvement, despite the fact that performance remained below 100%.

The population average curve is shown in **Figure 3B**. Every subject showed a monotonic, saturating improvement in accuracy with reaction time, but the reaction time distributions and accuracy varied from subject to subject. For each rat, trials with reaction times in that rat’s lowest quartile were defined as “fast,” and trials with reaction time in the rat’s highest quartile were defined as “slow.” Every rat performed better in slow trials than fast ones (**Figure 3C**); this improvement with reaction time was significant for 10/12 rats individually (the 95% binomial confidence intervals do not overlap), and the effect was significant at the population level (*P* < 10^-^^3^, Wilcoxon signed rank test).

### RATS TAKE MORE TIME TO RESPOND WHEN IMAGES ARE MORE SIMILAR

To test whether rats take longer to make a decision when the sensory stimuli are more ambiguous, the similarity of the two images was parametrically varied in probe trials with morphed images (see Materials and Methods; **Figure 1C**; Clark et al., 2011). Exemplar and morph trials were randomly interleaved in the experiment, but exemplar trials were far more numerous (see Materials and Methods).

Accuracy of discrimination decreased as the images became more similar, as shown for one rat in **Figure 4A** and summarized for all rats in **Figure 4B**. For the subject whose performance is shown in **Figure 4A**, the distribution of response latencies was shifted to longer latencies in the trials with more ambiguous stimuli (**Figure 4C**), indicating that this subject took more time on more difficult trials. For most subjects (*N* = 10/12 rats), the median reaction time on the easiest trials (exemplar, level 1) was lower than the median reaction time on the most difficult or ambiguous trials (morph levels 12–15; **Figure 4D**), and this trend was significant at the population level (*P* < 10^-^^2^, Wilcoxon signed rank test).

### RATS TAKE MORE TIME TO RESPOND WHEN THE ERROR PENALTY IS INCREASED

For two rats, we also compared reaction times and accuracy in paired testing blocks differing only in penalty duration (2 vs. 6 s). For both rats, increasing the duration of the error penalty led to a significant increase in DT (**Figure 5A**). This was accompanied by a substantial improvement in accuracy (**Figure 5B**), and therefore a lower probability of incurring the penalty. One rat (black lines) was tested with the exemplar discrimination pair described above. The other (gray lines) was tested using a more difficult discrimination pair (box/car image pair), after having trained to asymptotic performance of 65% on that discrimination. Incidentally, this second subject did not have longer reaction times on harder trials when they were interleaved (symbol below diagonal in **Figure 4D**; median DT 0.375 s for easy, 0.360 s for hard, penalty duration 2 s). Nevertheless, in an extended testing block with only difficult trials, reaction time was longer (median DT 0.630 at 2 s penalty duration) than in the easier discrimination block. Thus the subject did modulate reaction time with difficulty on the block timescale, even with penalty held constant.

Increasing the penalty duration led to a reduction in fast responses (0.5–1 s latency), and an increase in slow responses (1.0–1.5 s latency), for both rats (**Figures 6A–D**). Regardless of penalty condition, responses were rarely as fast as the rat’s estimated motor delay (vertical lines in **Figures 6A–D**). For the subject that was tested with a more difficult discrimination pair (gray in **Figure 5**; **Figures 6B,D**), performance was only 65% with the short penalty. Thus penalty was incurred in 45% of trials, substantially limiting reward rate. This rat’s reaction times shifted more dramatically in response to penalty increase.

## DISCUSSION

These data demonstrate an interaction between reaction time and accuracy in the visual discrimination of images of natural objects by rats. Rats performed better when they responded later (**Figures 2** and **3**), despite the absence of any temporal information in the stimulus itself. Moreover, most rats responded more slowly when confronted with more difficult discriminations (**Figure 4**), or when the cost of an error was higher (**Figures 5** and **6**).

### ACCURACY INCREASES WITH REACTION TIME

When rats discriminate static visual images without a deadline, their discrimination accuracy for a given discrimination difficulty improves with reaction time (**Figures 2** and **3**). The reaction times, accuracy, and dependence of accuracy on time, were all comparable to those reported for discrimination of random dot motion stimuli under similar conditions (Reinagel, 2013). In the random dot motion task, stimuli are rendered difficult both by reducing signal (fewer dots contributing to coherent motion) and adding noise (more dots moving randomly). In such stimuli, new sensory evidence is presented continuously over time, and temporal integration should improve signal-to-noise ratio. In our task, stimuli are rendered difficult by making them more similar (**Figure 1C**). The generalization to static images shows that the improvement in accuracy with time is not specific to temporally evolving visual stimuli, nor restricted to tasks with noise corruption in the physical stimulus. In our task, errors for very difficult morphs may be due to failure to perceive differences, but could also arise from a noisy category boundary.

We hypothesize that accuracy is determined by the amount of sensory evidence accumulated at the time the rat decides, regardless of what determines the time of the decision. In the case of motion discrimination this hypothesis was tested by uncoupling reaction time from viewing time (Reinagel, 2013), but the equivalent experiment has not been done for the image task.

In a related image discrimination task performed by rats (Zoccolan et al., 2009), accuracy was higher in the trials with short reaction times (Tafazoli et al., 2012). This seemingly opposite result was explained by priming effects in their experiment, however. In trials with congruent primes, rats were both faster and more accurate. The results reported here are not in conflict with that finding.

Our findings are also consistent with results from mice in a 2AFC auditory discrimination task (Sanders and Kepecs, 2012). In that task, like the random dot motion task, the stimulus unfolded over time and the signal was stochastic, such that optimal performance requires evidence accumulation. Accuracy increased with reaction time for easy discriminations, and reaction time increased with discrimination difficulty, as we found for visual tasks in rats. In that study, monitoring behavior during the decision interval revealed that mice make choice reversals that improve accuracy. Choice reversal could explain a correlation between accuracy and long reaction times in their task and in ours. We have no data, however, on the location or locomotion of the rats during the decision interval.

When primates perform visual reaction time tasks with interleaved trials of varying sensory difficulty, accuracy is widely reported to decline as a function of reaction time – the opposite of our result (Roitman and Shadlen, 2002; Mazurek et al., 2003; Palmer et al., 2005; Churchland et al., 2008). In those data this result is attributed to a collapsing decision bound, which can be explained in terms of accumulation of evidence during the decision interval about the quality of the sensory evidence in that the trial (Hanks et al., 2011; Huang et al., 2012). We still do not know if task differences, species differences, or both underlie these different experimental findings. The most obvious task difference is that we imposed no minimum response delay, no additional reward delays, and no minimum inter-trial interval in our task. Such enforced delays are typically used in the primate studies to discourage fast guessing, and have the consequence that DT is a small fraction of total trial time. Our task makes the cost of DT significant to the rate of reward harvesting, a regime that is not well explored in the speed–accuracy literature. Yet from the point of view of the animal, fast guessing is a valid reward harvesting strategy that may be optimal under some conditions. It will be interesting to develop quantitative models that include and account for this basic choice behavior.

### DETERMINANTS OF REACTION TIME

Using morphing to vary image discrimination difficulty, we found that rats responded later on more difficult trials (**Figure 4**). A similar result was found for rats in a random dot motion task (Reinagel, 2013). In a transformation-invariant visual object recognition task (Zoccolan et al., 2009), it has also been noted that reaction times are longer on more extreme transformations (Tafazoli et al., 2012; Alemi-Neissi et al., 2013). Accuracy decreased with difficulty while reaction time increased, consistent with our findings. In that task as in ours, discrimination difficulty was varied but the stimulus did not unfold over time or contain stochastic noise.

Although reaction time increased with difficulty in our task, the increase was modest – only about 100 ms on the most difficult trials. The difference in reaction time may reflect the lower confidence of the animal in hard trials (Kepecs et al., 2008; Kiani and Shadlen, 2009) rather than an accumulation of evidence strategy. One explanation for the rats’ failure to wait longer could be that rats lack the capacity to control impulsivity to optimize reward rate.

But here we report that rats can modulate their behavioral strategy in response to the cost of errors. When the duration of penalty was increased, rats waited longer before responding, and their accuracy improved (**Figures 5** and **6**). This is consistent with the idea that longer viewing time leads to more accurate discriminations. But it is equally possible that a third cause (such as increased attention) caused an increase in both reaction time and accuracy.

### SOURCE OF TIME-DEPENDENCE

The results presented here provide evidence for a time-dependent improvement in image discrimination, despite the absence of dynamics or time-varying noise in the stimulus. Because the physical stimulus was unchanging, this implies some temporal process arising in the animal. Possibilities are numerous and include: variation in the animal’s state (e.g., attention, motivation, or arousal) from trial to trial; active sampling of the visual stimulus (e.g., saccades, involuntary eye movements, head or body movements), sensory neural processing (e.g., temporal integration of noisy firing rates, spike time pattern codes), or cognitive processing involved in decision *per se*. The data presented here do not distinguish among these alternatives.

In particular, we do not know what the animal is doing, or when the decision occurs, within the interval between stimulus onset and detected response. If we had detected removal of the rat’s nose from the center port, this would have provided additional information, but we still would not know whether or when the rat made a decision until a response was made. A task in which motor output is monitored continuously could provide more insight into the time of the decision, including decision reversals within this interval (Sanders and Kepecs, 2012).

### GENERALITY OF FINDINGS

For the image discrimination task described here, we have shown that rats’ accuracy increases with reaction time, and reaction time is longer on harder stimuli, consistent with results from rats and mice tested with other visual and auditory stimuli, as summarized above. Nevertheless, these results may not be true for all sensory discrimination tasks. Clearly changes to the reward, penalty, or delay schedule of a task are expected to manipulate the relative priority of accuracy vs. speed. The relationship between reaction time and accuracy may also depend on the difficulty of the sensory discrimination, the sensory modality, or the qualitative nature of the sensory decision being made. In olfaction, for example, rats’ discrimination accuracy improves with reaction time in some tasks but not others (Uchida and Mainen, 2003; Abraham et al., 2004; Rinberg et al., 2006; Uchida et al., 2006). A complete theory of decision making will ideally encompass and account for such differences between tasks.

## Conflict of Interest Statement

The author declares that the research was conducted in the absence of any commercial or financial relationships that could be construed as a potential conflict of interest.

## AUTHOR CONTRIBUTIONS

The behavioral training protocol and visual task are from a previously published study (Clark et al., 2011), in which the performance of these same rats was already described without consideration of reaction time. Pamela Reinagel conceived of the present study, collected these additional reaction time data, analyzed the data, interpreted the results, and wrote this manuscript.

## SUPPLEMENTARY MATERIAL

The Supplementary Material for this article can be found online at http://www.frontiersin.org/Journal/10.3389/fncir.2013.00200/abstract
